# Estimation of material parameters based on precipitate shape: efficient identification of low-error region with Gaussian process modeling

**DOI:** 10.1038/s41598-019-52138-0

**Published:** 2019-10-31

**Authors:** Yuhki Tsukada, Shion Takeno, Masayuki Karasuyama, Hitoshi Fukuoka, Motoki Shiga, Toshiyuki Koyama

**Affiliations:** 1 Department of Materials Design Innovation Engineering, Graduate School of Engineering, Nagoya University, Furo-cho, Chikusa-ku, Nagoya 464-8603 Japan; 2 JST, PRESTO, 4-1-8 Honcho, Kawaguchi, Saitama 332-0012 Japan; 3 Department of Computer Science, Graduate School of Engineering, Nagoya Institute of Technology, Gokiso-cho, Showa-ku, Nagoya 466-8555 Japan; 4 Center for Materials Research by Information Integration, National Institute for Materials Science, 1-2-1 Sengen, Tsukuba, Ibaraki 305-0047 Japan; 5 Department of Electrical, Electronic and Computer Engineering, Faculty of Engineering, Gifu University, 1-1 Yanagido, Gifu, 501-1193 Japan; 6 Center for Advanced Intelligence Project, RIKEN, 1-4-1 Nihonbashi, Chuo-ku, Tokyo 103-0027 Japan

**Keywords:** Coarse-grained models, Computational methods

## Abstract

In this study, an efficient method for estimating material parameters based on the experimental data of precipitate shape is proposed. First, a computational model that predicts the energetically favorable shape of precipitate when a *d*-dimensional material parameter (*x*) is given is developed. Second, the discrepancy (*y*) between the precipitate shape obtained through the experiment and that predicted using the computational model is calculated. Third, the Gaussian process (GP) is used to model the relation between *x* and *y*. Finally, for identifying the “low-error region (LER)” in the material parameter space where *y* is less than a threshold, we introduce an adaptive sampling strategy, wherein the estimated GP model suggests the subsequent candidate *x* to be sampled/calculated. To evaluate the effectiveness of the proposed method, we apply it to the estimation of interface energy and lattice mismatch between MgZn_2_ (${{\rm{\beta }}}_{1}^{\text{'}}$) and α-Mg phases in an Mg-based alloy. The result shows that the number of computational calculations of the precipitate shape required for the LER estimation is significantly decreased by using the proposed method.

## Introduction

Precipitate shape in materials intrinsically contains some information on material parameters. The interaction between the interface and strain energies determines the equilibrium shape of a coherent precipitate^1–6^. For example, the aspect ratio of a plate- or rod-shaped coherent precipitate, often observed in Mg-based alloys^7–27^, varies depending on the precipitate size, interface energy, and crystal lattice mismatch between the precipitate and matrix phases. In materials science, material parameters have often been estimated by using experimental data. For example, the interface energy can be estimated by fitting the Ostwald ripening model^28^ (theoretical formula) to time-series experimental measurement of precipitate radius. Most recently, there are some efforts to estimate material parameters by comparing data of microstructure evolution obtained by experiment and simulation^29–32^. However, the material parameter estimation by directly using experimental data of precipitate shape has not been reported yet. This is due to the fact that although there are some computational models to predict precipitate shape^6,33–35^, their computational cost is high and hence it is time-consuming to estimate material parameters by fitting the models to experimental data.

A precipitate prefers an energetically favorable shape that minimizes the total energy derived from the precipitation. When a spheroidal precipitate is assumed, the total energy (sum of the interface and strain energies) can be calculated as a function of the aspect ratio of the spheroid (precipitate shape) for the given values of material parameters and precipitate volume^6^. Hence, the material parameters involved in the total energy calculation can be estimated if the experimental data of the precipitate shape are obtained. However, considering that the computational cost of the total energy calculation is high and the number of candidate parameter conditions is large, an efficient method for material parameter estimation is necessary. Given that experimental data of precipitate shape are naturally uncertain, estimating the “*low-error region* (LER)” in the material parameter space seems to be crucial, where the discrepancy between the precipitate shape obtained through the experiment and that predicted using a computational model becomes small.

In this study, we propose an efficient method for estimating material parameters based on the experimental data of precipitate shape. First, a computational model that predicts the energetically favorable shape of the precipitate under a given material parameter condition is developed. When the *d*-dimensional material parameter (${\boldsymbol{x}}\in {{\mathbb{R}}}^{d}$) is given, we can calculate the discrepancy (*y*) between the precipitate shape obtained through the experiment and that predicted using the computational model. Then, we use the Gaussian process (GP) to model the ***x*** and *y* relation. GP has been widely used for a variety of problems in materials science such as materials discovery^36^, potential approximation^37^, and structure optimization^38,39^. Unlike classical deterministic regression models, GP represents an unknown target function value as a random variable of a Gaussian distribution, which enables us to simply quantify uncertainty of the current prediction. We utilized this uncertainty evaluation to define probabilistic estimation of LER for each uncalculated candidate ***x***. Finally, we introduce an adaptive sampling strategy wherein the estimated GP model suggests the subsequent candidate ***x*** to be sampled/calculated so that the uncertainty of the LER estimation is efficiently reduced. Since our interest is only in LER, exhaustive sampling in the parameter space should be inefficient. Our strategy intensively selects samples which are effective for identifying LER efficiently, instead of trying to approximate the entire discrepancy surface precisely. The result showed that the proposed method can provide an efficient estimation of material parameters.

## Results

To evaluate the effectiveness of the proposed method, we consider estimating the interface energy and lattice mismatch between the hexagonal MgZn_2_ (${{\rm{\beta }}}_{1}^{\text{'}}$) precipitate and hexagonal α-Mg matrix phases based on the experimental data on the shape of the ${{\rm{\beta }}}_{1}^{\text{'}}$ phase. The stress-free transformation strain (crystal lattice mismatch between the ${{\rm{\beta }}}_{1}^{\text{'}}$ and α phases) is expressed by

1 $${\varepsilon }_{ij}^{0}=(\begin{array}{ccc}{\varepsilon }_{11}^{0} & 0 & 0\\ 0 & {\varepsilon }_{22}^{0} & 0\\ 0 & 0 & {\varepsilon }_{33}^{0}\end{array}).$$

**Figure 1:**
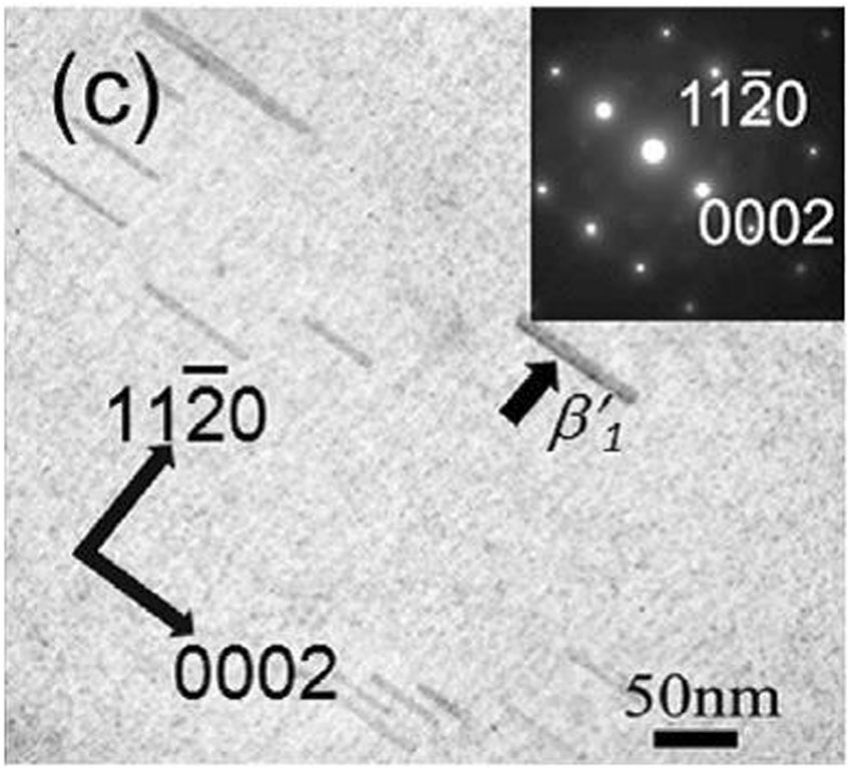
Shape of the ${{\rm{\beta }}}_{1}^{\text{'}}$ phase investigated via transmission electron microscopy in Mg–Zn–Ca–Ag alloy, which is aged at 160 °C for 2 h. (Reprinted from Mater. Sci. Eng. A, Vol. 575, Bhattacharjee, T., Mendis, C.L., Oh-ishi, K., Ohkubo, T. & Hono, K., The effect of Ag and Ca additions on the age hardening response of Mg–Zn alloys, pp. 231–240, 2013, with permission from Elsevier).

Here, ${\varepsilon }_{11}^{0}$, ${\varepsilon }_{22}^{0}$, and ${\varepsilon }_{33}^{0}$ are the lattice mismatch along ${[2\bar{1}\bar{1}0]}_{{\rm{\alpha }}}$, ${[01\bar{1}0]}_{{\rm{\alpha }}}$, and ${[0001]}_{{\rm{\alpha }}}$ of the α phase, respectively. Figure 1 shows the shape of the ${{\rm{\beta }}}_{1}^{\text{'}}$ phase observed in an aged Mg–Zn–Ca–Ag alloy^24^. The ${{\rm{\beta }}}_{1}^{\text{'}}$ phase has a rod shape along [0001]_α_. Hence, the values of ${\varepsilon }_{11}^{0}$ and ${\varepsilon }_{22}^{0}$ are assumed to be equal^6^. Based on the experimental data on the crystallographic orientation relationship between the ${{\rm{\beta }}}_{1}^{\text{'}}$ and α phases^8^ and lattice parameters of the two phases^18,40^, the lattice mismatch along [0001]_α_ is ${\varepsilon }_{33}^{0}=0.00182$. Table 1 presents the change in the length and diameter of the rod-shaped ${{\rm{\beta }}}_{1}^{\text{'}}$ phase during aging at 160 °C measured via transmission electron microscopy (TEM)^24^. By using the experimental data listed in the table, we consider estimating the interface energy *γ* and the lattice mismatch ${\varepsilon }_{11}^{0}(\,=\,{\varepsilon }_{22}^{0})$.

**Table 1:** Shape change of the rod-shaped MgZn_2_ (${{\rm{\beta }}}_{1}^{\text{'}}$) phase during aging at 160 °C in Mg–Zn–Ca–Ag alloy^24^.

Aging time (*t*/h)	Length (nm)	Diameter (nm)	Average of aspect ratio (*r*_expt_)
2	60.5 ± 14.0	5.9 ± 1.3	10.25 ± 0.14
8	62.6 ± 21.6	6.1 ± 1.4	10.26 ± 1.53
24	85.0 ± 28.3	7.3 ± 1.4	11.64 ± 2.02

**Figure 2:**
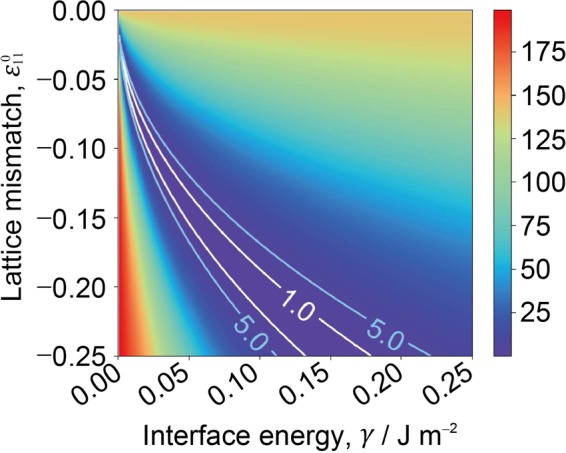
Heatmap of *y*_*i*_, which is the error in the aspect ratio between the precipitate obtained through the experiment and that predicted using the computational model. Low-error region (LER), where *y*_*i*_ ≤ *h*, is shown for *h* = 1 and *h* = 5 as denoted by white and blue lines, respectively.

We assume that the ${{\rm{\beta }}}_{1}^{\text{'}}$ phase has a spheroidal shape. Then, we prepare the computational model for predicting the energetically favorable shape of the precipitate (aspect ratio of the spheroid) with the given values of *γ* and ${\varepsilon }_{11}^{0}$ (refer to “Methods” section for details). To determine the candidate parameter ${\boldsymbol{x}}={(\gamma ,{\varepsilon }_{11}^{0})}^{{\rm{\top }}}$, we use 250 equally spaced grids in ${\varepsilon }_{11}^{0}\in [-\,0.250,-\,0.001]$ and 250 equally spaced grids in *γ*∈[0.001,0.250] (J m^−2^). Thus, we have a total of 62500 candidate parameter conditions ***x***_*i*_(*i* = 1, …, 62500). In the given material parameter condition ***x***_*i*_, the discrepancy in the aspect ratio between the precipitate obtained through the experiment (*r*_expt_) and that predicted using the computational model (*r*_*i*,comput_) is defined as follows:

2 $${y}_{i}=\frac{1}{2}\sum _{t\in {\mathscr{T}}}\,{({r}_{{\rm{expt}}}(t)-{r}_{i,{\rm{comput}}}(t))}^{2},$$

where *t* is time and ${\mathscr{T}}$ is a set of time when the shape of ${{\rm{\beta }}}_{1}^{\text{'}}$ is experimentally measured. We consider identifying the LER where the error *y*_*i*_ is less than a given threshold *h*, i.e., *y*_*i*_ ≤ *h*. Based on the standard deviation of *r*_expt_ listed in Table 1, *h* is assumed to have a value between 1 and 5. Figure 2 shows the heatmap of *y*_*i*_ in the material parameter space, in which the LER for *h* = 1 and *h* = 5 are denoted by white and blue lines, respectively.

GP is used to model the relation between ***x***_*i*_ and *y*_*i*_. In this process, *y*_*i*_ is modeled as *y*_*i*_ = *f*_*i*_ + *e*, where $e \sim {\mathscr{N}}(0,\varepsilon )$ is an independent noise term with variance *ε*. We assume that the noise is negligible because it is associated with the numerical error of the computational model. Thus, we set a small value of *ε*, as will be described in “Methods” section. Then, the conditional distribution for *f*_*i*_ after observing ${{\boldsymbol{y}}}_{{\mathscr{I}}}$ (a vector defined by *y*_*i*_ for $i\in  {\mathcal I}$, where ${\mathcal I} \subseteq \{1,\cdots ,N\}$ is a subset of indices for which *y*_*i*_ is already calculated) is expressed as

3 $${f}_{i}\,|\,{{\boldsymbol{y}}}_{{\mathscr{I}}}\sim {\mathscr{N}}(\mu ({{\boldsymbol{x}}}_{i}),{\sigma }^{2}({{\boldsymbol{x}}}_{i})),$$

where *μ*(***x***_*i*_) and *σ*(***x***_*i*_) are the conditional mean and covariance functions, respectively. In the proposed method, the estimated GP model suggests the subsequent candidate ***x***_*i*_ that has the maximum *information gain* (IG) among the uncalculated points (refer to “Methods” section for details). The intuition behind IG is to add a sample which has the highest uncertainty reduction among candidates. Our sampling strategy can be summarized as follows:

- fit GP to the observed ${{\boldsymbol{y}}}_{{\mathscr{I}}}$, and obtain the probabilistic estimation of LER, and
- select the candidate ***x***_*i*_ which has the highest IG evaluated through the fitted GP.

For the material parameter ***x***_*i*_, we use the indicator variables as follows:

4 $${z}_{i}=\{\begin{array}{cc}0,\,{\rm{i}}{\rm{f}} & {f}_{i} > h,\\ 1,\,{\rm{i}}{\rm{f}} & {f}_{i}\le h.\end{array}$$

**Figure 3:**
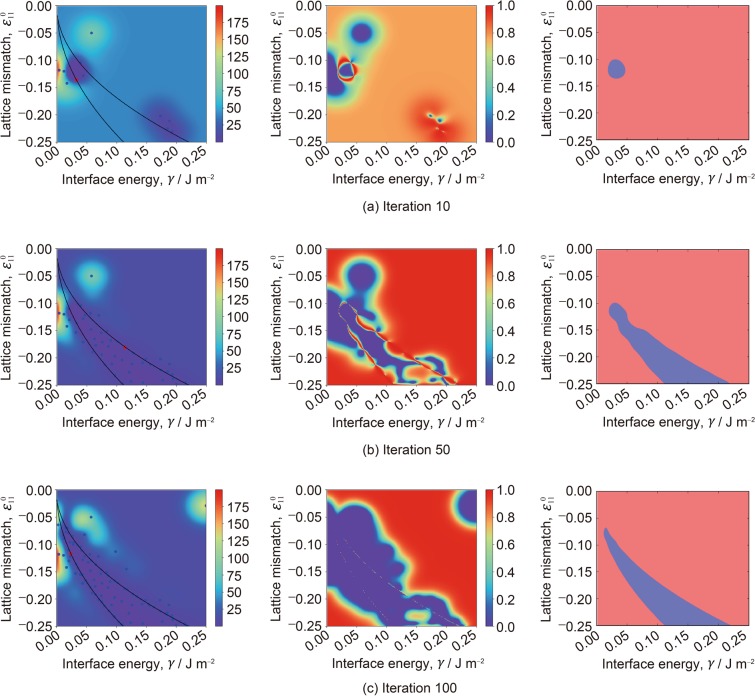
Demonstration of the proposed sampling strategy with *h* = 5. The images on the left column are the heatmap of *μ*(***x***_*i*_). Blue points represent the calculated points, and the red point is the one to be calculated in the subsequent iteration. Black lines are the boundary of the LER for *h* = 5. The images at the middle column are the heatmap of IG. The images on the right column are the predicted LER. Blue regions are *p*(*z*_*i*_ = 1) ≥ 0.5, and red regions are *p*(*z*_*i*_ = 1) < 0.5. In each iteration, a candidate ***x*** which has the highest IG is sampled. Thus, the number of samples in each plot (a), (b), and (c) is equal to the number of iterations.

Figure 3 shows the sampling process of the proposed method. In the figure, the left, middle, and right columns are the heatmap of *μ*(***x***_*i*_), heatmap of IG, and predicted LER (blue regions are *p*(*z*_*i*_ = 1) ≥ 0.5, where *p* represents the probability of *z*_*i*_), respectively. Note that it is known that *μ*(***x***) is equivalent to the well-known non-parametric regression model called *kernel ridge regression* (KRR)^41^. The non-parametric approach is suitable to our problem setting than the parametric approach such as classical linear regression. This is because, in the parametric approach, the discrepancy *y*_*i*_ has to be modeled as an explicit function of material parameters, but the functional form of the discrepancy surface is not known beforehand. In the left columns in Fig. 3, the blue points are the sampled points, red point is the point to be sampled in the subsequent iteration, and black lines are the boundary of the LER for *h* = 5. Although the error surface in iteration 10 remains highly uncertain, a part of the LER is already identified. In iteration 50, the entire shape of the LER is barely identified, and the sampled points are mostly concentrated in the LER. In iteration 100, the points around the LER boundary are sampled to identify the region precisely.

**Figure 4:**
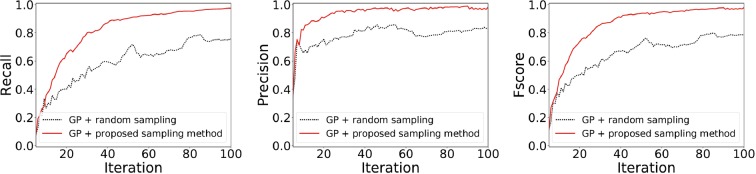
Performance evaluation with *h* = 5. The image on the left is the ratio of points *i* for which *p*(*z*_*i*_ = 1) ≥ 0.5 among the points in LER (recall). The image at the middle is the ratio of points *i* for which *y*_*i*_ ≤ *h* among the points in the predicted LER *p*(*z*_*i*_ = 1) ≥ 0.5 (precision). The image on the right is the harmonic mean of recall and precision (F-score).

Figure 4 shows the performance evaluation. Our purpose is to identify LER. This problem setting can be seen as a variant of *classification problem* in which the binary label *z*_*i*_ is needed to be predicted precisely. Thus, we evaluate accuracy of the LER prediction by *z*_*i*_ by using standard evaluation measures of classification problem. We use three criteria, namely *recall*, *precision*, and *F-score*, that are extensively used in the field of *information retrieval*^42^. Each plot compares the performance of two GP models, that is, (1) GP with our sampling strategy and (2) GP with random sampling. For both sampling approaches, a candidate ***x*** is classified as LER if *p*(*z*_*i*_ = 1) ≥ 0.5, which is equivalent to *μ*(***x***_*i*_) ≤ *h*. Since *μ*(***x***_*i*_) is equivalent to KRR, “GP + random sampling” can also be seen as a baseline defined by KRR with a naive sampling strategy. The left plot in Fig. 4 shows the recall, defined by

$$\frac{{\rm{The}}\,{\rm{number}}\,{\rm{of}}\,{\rm{points}}\,i\,{\rm{for}}\,{\rm{which}}\,p({z}_{i}=1)\ge 0.5\,{\rm{and}}\,{y}_{i}\le h}{{\rm{The}}\,{\rm{number}}\,{\rm{of}}\,{\rm{points}}\,i\,{\rm{for}}\,{\rm{which}}\,{y}_{i}\le h}.$$

Thus, the recall is the ratio of the number of LER points that GP correctly identifies over the number of points in true LER. This can evaluate how many LER points are correctly identified. At the beginning of the iterations, the recall was approximately 0.1 for both sampling strategies owing to the absence of sampled points. However, we observe that the proposed method rapidly increased the recall substantially faster than the random sampling. The middle plot in Fig. 4 shows the precision, defined by

$$\frac{{\rm{The}}\,{\rm{number}}\,{\rm{of}}\,{\rm{points}}\,i\,{\rm{for}}\,{\rm{which}}\,p\,({z}_{i}=1)\ge 0.5\,{\rm{and}}\,{y}_{i}\le h}{{\rm{The}}\,{\rm{number}}\,{\rm{of}}\,{\rm{points}}\,i\,{\rm{for}}\,{\rm{which}}\,p({z}_{i}=1)\ge 0.5}.$$

The precision has the same numerator as recall, but the denominator is the number of points predicted as LER by GP. This can evaluate specificity of the prediction, which cannot be considered by recall. Considering that most of the predicted LER was actually *y*_*i*_ ≤ *h* (Fig. 3), the precision values are higher than the recall from the beginning of the iterations. Hence, the proposed sampling strategy is substantially better than the random sampling strategy. Given that recall and precision sometimes have a tradeoff relationship, their harmonic mean, referred to as F-score, is often used as the basis for a comprehensive evaluation. Figure 4 (right plot) shows the superior performance of the proposed method in F-score. The effectiveness of the proposed method was similarly evaluated when we set *h* = 1. The proposed method identified the LER accurately after 100 iterations despite the region was narrower than that of *h* = 5 (refer to Supplementary Information).

## Discussion

Prior to material parameter estimation, determining the material parameter range of interest is crucial. In this study, the parameter ranges of ${\varepsilon }_{11}^{0}$ and *γ* were assumed to be ${\varepsilon }_{11}^{0}\in [\,-\,0.250,-\,0.001]$ and $\gamma \in [0.001,0.250]$ (J m^−2^), respectively. When the ${{\rm{\beta }}}_{1}^{\text{'}}$ phase grows during aging heat treatment, the interface between ${{\rm{\beta }}}_{1}^{\text{'}}$ and α phases would shift from coherent to semi-coherent/incoherent. As TEM micrographs^24^ show, the α/${{\rm{\beta }}}_{1}^{\text{'}}$ interface in the Mg–Zn–Ca–Ag alloy remains coherent during aging at 160 °C for 2–24 h because any dislocations do not occur at the interface. The coherent interface energy is generally less than 0.25 J m^−2^ (ref.^5^). Hence, the parameter range of $\gamma \in [0.001,0.250]$ (J m^−2^) is reasonable. Furthermore, the absolute value of ${\varepsilon }_{11}^{0}$ larger than 0.25 was omitted from the parameter range of interest because this value was much larger than the reported values of the lattice mismatch between precipitate and matrix phases in several Mg-based alloys^33–35^.

The lattice mismatch between precipitate and matrix phases is often estimated from the lattice parameter information of the two phases and crystallographic lattice correspondence between the two phases^4,33–35^. However, regarding the nanometer-size precipitate, experimentally determining the lattice correspondence using TEM is challenging, specifically when the aspect ratio of the precipitate is high/low. Moreover, the aspect ratio of the nanometer-size ${{\rm{\beta }}}_{1}^{\text{'}}$ phase is higher than 10 (Table 1). Hence, the lattice correspondence between ${{\rm{\beta }}}_{1}^{\text{'}}$ and α phases along the crystallographic directions perpendicular to [0001]_α_ is difficult to determine experimentally. In contrast, our method is based on the computational model for predicting the energetically favorable shape of precipitate with the given material parameters. The method can perform simultaneous estimation of the lattice mismatch and interface energy based on the experimental data of precipitate shape. Furthermore, it only requires at most 100 computational calculations of the precipitate shape to estimate the LER. Thus, we assume that the proposed method can be used for the effective utilization of the experimental data of precipitate shape for estimating material parameters.

The computational model used in predicting the energetically favorable shape of precipitate can be replaced by another computational model or simulation if necessary. The limitation of the computational model used in this study is that the precipitate shape is assumed to be spheroid and the interface energy anisotropy is ignored (refer to “Methods” section for details). In several Mg-based alloys^43–46^, the precipitate mostly has faced or lenticular shape, on which the anisotropy of the interface energy has significant effects^34^. Furthermore, although we assume that the precipitate takes on an energetically favorable shape, there would be some cases where the precipitate shape cannot be fully accommodated during aging. In the above-mentioned cases, the computational model used in this study should be replaced by a phase-field model^33–35^, which can simulate the microstructure evolution that takes place so as to reduce the total free energy of the microstructure^47,48^. The computational cost of the phase-field simulation in predicting precipitate shape change would be much higher than the computational model employed in this study. However, it is presumed that combining the phase-field and GP models with our sampling strategy would also provide an efficient estimation of material parameters based on the precipitate shape. Note that the material parameter estimation primarily from the precipitate shape is challenging in the case where the precipitate volume fraction is high and long-range interaction between the precipitates is strong because the precipitate shape is determined by not only the material parameters but also the precipitate spatial arrangement.

In summary, if a computational model for predicting the energetically favorable shape of precipitate with the given material parameters is available, it is possible to estimate material parameters based on the precipitate shape. The GP model combined with our sampling strategy can significantly reduce the computational cost required for the LER estimation and would be useful to accelerate the extraction of material parameters from experimental data of precipitate shape obtained at various temperatures in different alloys.

## Methods

### Prediction of the precipitate aspect ratio

When a spheroidal precipitate is assumed (*x*^2^/*a*^2^ + *y*^2^/*b*^2^ + *z*^2^/*c*^2^ = 1, *a* = *b*, *r* = *c*/*a*), the total energy (sum of the interface and strain energies) of the precipitate is formulated as a function of the aspect ratio of the spheroid *r* (ref.^6^).

5 $$\begin{array}{rcl}{E}_{{\rm{total}}}(r) & \equiv  & {E}_{{\rm{strain}}}(r)+{E}_{{\rm{interface}}}(r)\\  & = & \frac{{V}_{0}}{2}{C}_{ijkl}{\varepsilon }_{kl}^{0}({\varepsilon }_{ij}^{0}-{S}_{ijmn}(r){\varepsilon }_{mn}^{0})+A(r)\gamma ,\end{array}$$

where *V*_0_ is precipitate volume, *C*_*ijkl*_ is elastic modulus tensor, ${\varepsilon }_{ij}^{0}$ is crystal lattice mismatch between the precipitate and matrix phases, *S*_*ijmn*_(*r*) is Eshelby’s tensor^49^, *A*(*r*) is interface area, and *γ* is interface energy (in J m^−2^). Note that anisotropy of the interface energy is ignored for simplicity. The computational cost for calculating *S*_*ijmn*_(*r*) is high because the surface integration must be numerically calculated^6^.

**Figure 5:**
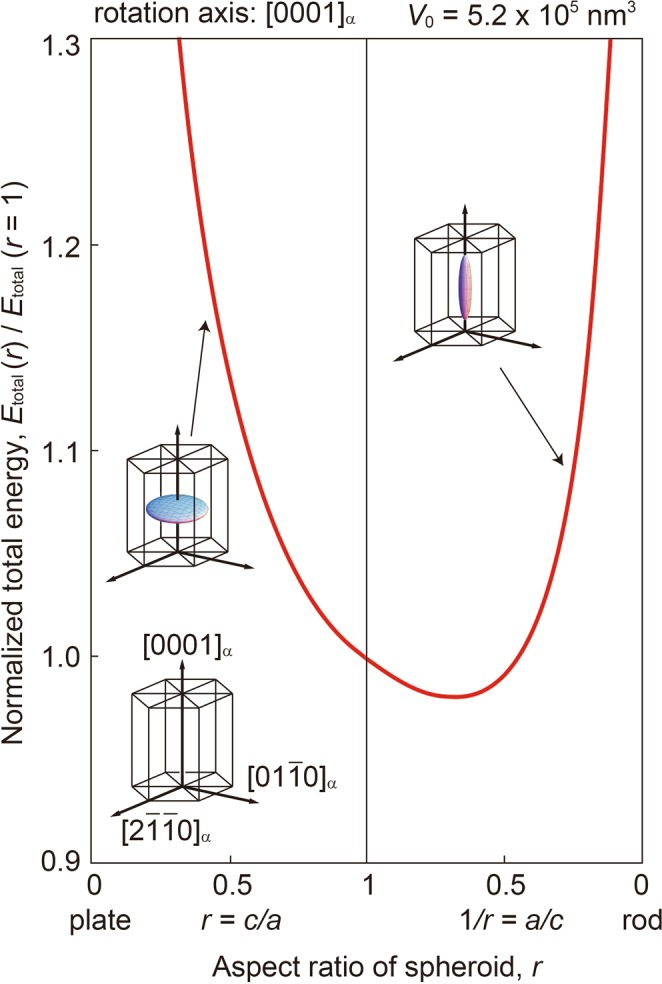
Total energy *E*_total_ (sum of the strain and interface energies) of the spheroidal precipitate as a function of the aspect ratio of spheroid *r*.

We consider predicting the aspect ratio of the ${{\rm{\beta }}}_{1}^{\text{'}}$ phase in the α phase with the given material parameters. ${\varepsilon }_{ij}^{0}$ is given by Eq. (1), where ${\varepsilon }_{11}^{0}={\varepsilon }_{22}^{0}$. As an example, Fig. 5 shows the total energy *E*_total_ given by Eq. (5) as a function of the aspect ratio of the spheroid *r*. The calculation uses the following variables: interface energy *γ* = 0.1 J m^−2^; lattice mismatch ${\varepsilon }_{11}^{0}={\varepsilon }_{22}^{0}=0.01$ and ${\varepsilon }_{33}^{0}=0.002$; elastic modulus *C*_11_ = *C*_22_ = 0.597, *C*_33_ = 0.617, *C*_12_ = 0.262, *C*_13_ = *C*_23_ = 0.217, *C*_44_ = *C*_55_ = 0.164, and *C*_66_ = (*C*_11_−*C*_12_)/2 (10^11^ Pa)^40^; and ${{\rm{\beta }}}_{1}^{\text{'}}$ phase volume *V*_0_ = 5.2 × 10^5^ nm^3^. The rotation axis of the spheroid is parallel to [0001]_α_ and *E*_total_ is normalized using *E*_total_ of the spherical precipitate (*r* = 1) with the same volume of ${{\rm{\beta }}}_{1}^{\text{'}}$ phase. As shown in the figure, the total energy is minimized when 1/*r* = 0.67, indicating that with given material parameters, we can compute the aspect ratio of the precipitate (*r*_comput_).

### GP model using selective sampling procedure

Suppose that ${{\boldsymbol{x}}}_{i}\in {{\mathbb{R}}}^{d}$ is a *d*-dimensional parameter of the computational model and ${y}_{i}\in {\mathbb{R}}$ is the discrepancy between the precipitate aspect ratio obtained through the experiment (*r*_expt_) and that predicted using the computational model (*r*_*i*,comput_) (Eq. (2)). Considering that the aspect ratio of the precipitates in the TEM micrograph fluctuates (Fig. 1), the average of the aspect ratio is used to calculate *y*_*i*_. To incorporate this intrinsic uncertainty in the experimental measurement, we consider identifying the LER, where *y*_*i*_ ≤ *h*. Compared with naturally searching a single optimal material parameter set, the LER provides the information as follows:

- the region of possible good material parameter sets that can be estimated from the experimental data, and
- the accuracy in identifying the material parameter sets from the current experimental measurement (large LER indicates that the error level *h* is substantially high for accurately identifying material parameter sets).

Considering that the computational cost of *y*_*i*_ (or *r*_*i*,comput_) is high, we estimate the LER according to the limited number of calculations of *y*_*i*_ based on a probabilistic model.

The relation between ***x***_*i*_ and *y*_*i*_ is modeled by GP. For *N* different parameter sets ***x***_*i*_(*i* = 1, …, *N*), the set of error values *y*_*i*_(*i* = 1, …, *N*) is approximated using the following multi-dimensional Gaussian distribution.

6 $${\boldsymbol{f}} \sim {\mathscr{N}}({\boldsymbol{\mu }},\,{\boldsymbol{K}}),$$

where ${\boldsymbol{f}}={({f}_{1},\cdots ,{f}_{N})}^{\top }$ and ${\mathscr{N}}({\boldsymbol{\mu }},\,{\boldsymbol{K}})$ is the Gaussian distribution with ${\boldsymbol{\mu }}\in {{\mathbb{R}}}^{N}$ as the mean vector and ${\boldsymbol{K}}\in {{\mathbb{R}}}^{N\times N}$ as the covariance matrix. The *i*,*j* element of the covariance matrix ***K*** is defined by the kernel function *k*(***x***_*i*_, ***x***_*j*_) for which we use the standard Gaussian kernel as follows:

7 $$k({{\boldsymbol{x}}}_{i},{{\boldsymbol{x}}}_{j})={\theta }_{1}\,\exp (-\frac{{\Vert {{\boldsymbol{x}}}_{i}-{{\boldsymbol{x}}}_{j}\Vert }^{2}}{2{\theta }_{2}^{2}}),$$

where *θ*_1_ and *θ*_2_ are the tuning parameters. Herein, the tuning parameters *θ*_1_ and *θ*_2_ in GP are optimized based on *marginal likelihood maximization*^41^ per iteration. By using the kernel function, the proximity of ***x***_*i*_ and ***x***_*j*_ is translated into the covariance of *f*_*i*_ and *f*_*j*_. Let ${\mathcal I} \subseteq \{1,\cdots ,N\}$ be the subset of indices for which *y*_*i*_ is already calculated and ${{\boldsymbol{y}}}_{{\mathscr{I}}}$ be the vector defined by* y*_*i*_ for $i\in  {\mathcal I}$. In this process, a calculated *y*_*i*_ is defined as *y*_*i*_ = *f*_*i*_ + *e*, where $e \sim {\mathscr{N}}(0,\varepsilon )$ is an independent noise term with variance *ε*. Herein, the variance *ε* is set to 10^−8^. The prior mean ***μ*** in Eq. (6) is set as the mean of observed *y*_*i*_ for $i\in  {\mathcal I}$. Then, the conditional distribution *f*or *f*_*i*_ after calculating ${{\boldsymbol{y}}}_{{\mathscr{I}}}$ is expressed in Eq. (3). ${f}_{i}\,|\,{{\boldsymbol{y}}}_{{\mathscr{I}}}$ is called the predictive distribution, and *μ*(***x***_*i*_*)* and *σ*(***x***_*i*_) in this equation are analytically written as follows:

8 $$\mu ({{\boldsymbol{x}}}_{i})={{\boldsymbol{K}}}_{i,{\mathscr{I}}}{({{\boldsymbol{K}}}_{{\mathscr{I}},{\mathscr{I}}}+\varepsilon {\boldsymbol{I}})}^{-1}({{\boldsymbol{y}}}_{{\mathscr{I}}}-{{\boldsymbol{\mu }}}_{{\mathscr{I}}}),$$

9 $${\sigma }^{2}({{\boldsymbol{x}}}_{i})=k({{\boldsymbol{x}}}_{i},{{\boldsymbol{x}}}_{j})-{{\boldsymbol{K}}}_{i,{\mathscr{I}}}{({{\boldsymbol{K}}}_{{\mathscr{I}},{\mathscr{I}}}+\varepsilon {\boldsymbol{I}})}^{-1}{{\boldsymbol{K}}}_{{\mathscr{I}},i},$$

where ***I*** is identity matrix. Moreover, the vectors and matrices with subscripts represent subvectors and submatrices specified by the given indices (e.g., ${{\boldsymbol{K}}}_{i,{\mathscr{I}}}$ indicates the *i*-th row and the columns defined by ${\mathscr{I}}$).

The indicator variable *z*_*i*_ defined by Eq. (4) represents whether the parameter ***x***_*i*_ is expected to have lower error than the threshold *h*. Given that *f*_*i*_ is the Gaussian random variable, the probability of *z*_*i*_ is simply expressed as follows:

10 $$p({z}_{i}=1)=\Phi (\frac{h-\mu ({{\boldsymbol{x}}}_{i})}{\sigma ({{\boldsymbol{x}}}_{i})}),$$

11 $$p({z}_{i}=0)=1-p({z}_{i}=1),$$

where Φ is the cumulative distribution function of the standard normal distribution. This probability provides a statistical estimation on the extent that the parameter ***x***_*i*_ has lower error than *h* based on the present calculations. Thus, a set of parameter that provides lower errors than *h* (i.e., LER) can be estimated as a set of ***x***_*i*_ satisfying the following equation.

12 $$p({z}_{i}=1)\ge \mathrm{0.5.}$$

Although GP provides the LER estimation, its reliability depends on the calculated points ${\mathcal I}$ (i.e., training data). To obtain an accurate prediction efficiently, we introduce an adaptive sampling strategy, referred to as *active learning*^50^, in which the estimated GP model suggests the subsequent candidate ***x***_*i*_ to be sampled/calculated to gain the prediction reliability. The basic idea is to select a point ***x***_*i*_ that can primarily reduce the uncertainty of *z*_*i*_. The following entropy evaluates the current uncertainty of *z*_*i*_.

13 $$H({z}_{i})=-\,\sum _{{z}_{i}\in \{0,1\}}\,p({z}_{i})\,\log \,p({z}_{i}).$$

The uncertainty of *z*_*i*_ after calculating *y*_*i*_ is represented by the conditional entropy as follows:

14 $$H({z}_{i}|{y}_{i})={{\mathbb{E}}}_{p({y}_{i}|{{\boldsymbol{y}}}_{ {\mathcal I} })}[\sum _{{z}_{i}\in \{0,1\}}\,p({z}_{i}|{y}_{i})\,\log \,p({z}_{i}|{y}_{i})].$$

Thus, by obtaining the difference between *H*(*z*_*i*_) and *H*(*z*_*i*_|*y*_*i*_), uncertainty reduction, referred to as IG, can be evaluated as follows:

15 $${{\rm{IG}}}_{i}=H({z}_{i})-\,H({z}_{i}|{y}_{i}).$$

The expectation in the second term *H*(*z*_*i*_|*y*_*i*_) is approximated by sampling from $p({y}_{i}|{{\boldsymbol{y}}}_{{\mathscr{I}}})$, defined by the Gaussian ${\mathscr{N}}(\mu ({{\boldsymbol{x}}}_{i}),{\sigma }^{2}({{\boldsymbol{x}}}_{i})+\varepsilon )$. Given the sample${\bar{y}}_{i}$ from $p({y}_{i}|{{\boldsymbol{y}}}_{{\mathscr{I}}})$, *f*_*i*_ is represented by ${\mathscr{N}}({\bar{y}}_{i},\varepsilon )$ because of our assumption that *y*_*i*_ = *f*_*i*_ + *e* (where $e \sim {\mathscr{N}}(0,\varepsilon )$). Then, we can easily calculate $p({z}_{i}=1\,|\,{\bar{y}}_{i})=p({f}_{i}\le h\,|\,{\bar{y}}_{i})$, which is equal to the cumulative distribution function of ${f}_{i}|{\bar{y}}_{i} \sim {\mathscr{N}}({\bar{y}}_{i},\varepsilon )$. We iteratively select the point ***x***_*i*_ that has the maximum IG_*i*_ among the uncalculated points.

**Figure 6:**
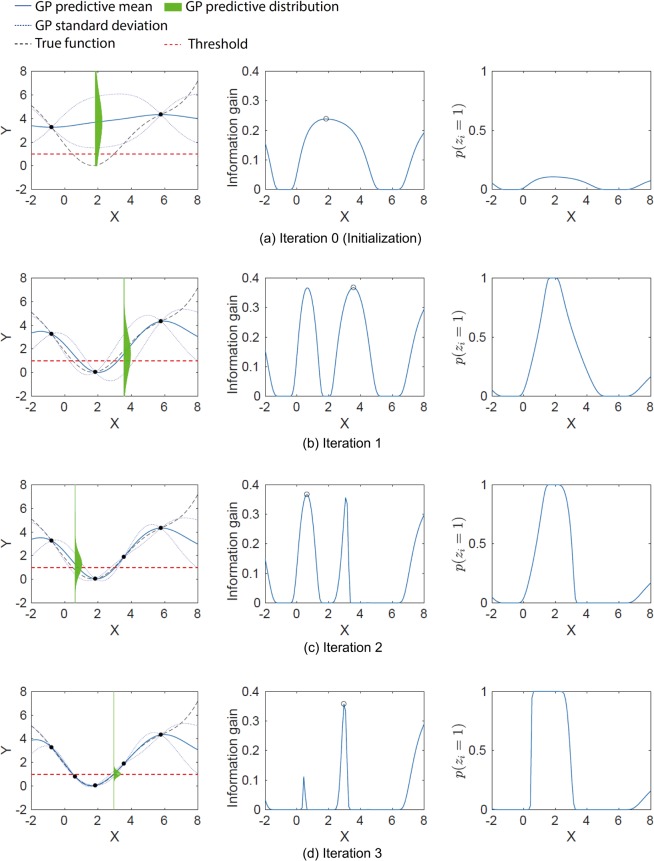
Illustrative example of the proposed method. The images on the left column are the prediction by GP. The predictive distribution is drawn at the point that has the maximum IG. The images at the middle are the IG, where the circle represents the maximum. The images on the right column are the probability of the LER: *p*(*z*_*i*_ = 1).

Figure 6 presents the illustrative example of the proposed method. The three plots in each iteration are the estimated GP model, IG, and *p*(*z*_*i*_ = 1). Our method iteratively selects a point that maximizes the IG per iteration, by which *p*(*z*_*i*_ = 1) gradually takes a value close to 1 or 0. Therefore, by sampling the point that reduces the prediction uncertainty, the GP model immediately increases its confidence on the LER identification. The proposed method can be generally applied to types of problem where the surface of *y* is smooth in ***x*** space.

## Supplementary information

Supplementary figures

## Data Availability

The Supplementary Information file provides the data supporting the results obtained in this study.
