# Supplementary material for: Estimation of material parameters based on precipitate shape: efficient identification of low-error region with Gaussian process modeling
Source: Sci Rep. 2019 Oct 31;9:15794. doi: 10.1038/s41598-019-52138-0 (PMC6823509; doi:10.1038/s41598-019-52138-0)
Supplement: Supplementary file 1 — Supplementary figures [file 41598_2019_52138_MOESM1_ESM.pdf]

Supplementary Information

Estimation of material parameters based on precipitate shape: efficient identification of low-error region with Gaussian process modeling

Yuhki Tsukada[1,2,*], Shion Takeno[3], Masayuki Karasuyama[2,3,4], Hitoshi Fukuoka[1], Motoki Shiga[2,5,6], Toshiyuki Koyama[1]

[1] Department of Materials Design Innovation Engineering, Graduate School of Engineering, Nagoya University, Furo-cho, Chikusa-ku, Nagoya 464-8603, Japan

[2] JST, PRESTO, 4-1-8 Honcho, Kawaguchi, Saitama 332-0012, Japan

[3] Department of Computer Science, Graduate School of Engineering, Nagoya Institute of Technology, Gokiso-cho, Showa-ku, Nagoya 466-8555, Japan

[4] Center for Materials Research by Information Integration, National Institute for Materials Science, 1-2-1 Sengen, Tsukuba, Ibaraki 305-0047, Japan

[5] Department of Electrical, Electronic and Computer Engineering, Faculty of Engineering, Gifu University, 1-1 Yanagido, Gifu 501-1193, Japan

[6] Center for Advanced Intelligence Project, RIKEN, 1-4-1 Nihonbashi, Chuo-ku, Tokyo 103-0027, Japan

[*]Corresponding author

Tel.: +81-52-789-3228

E-mail: tsukada.yuhki@material.nagoya-u.ac.jp

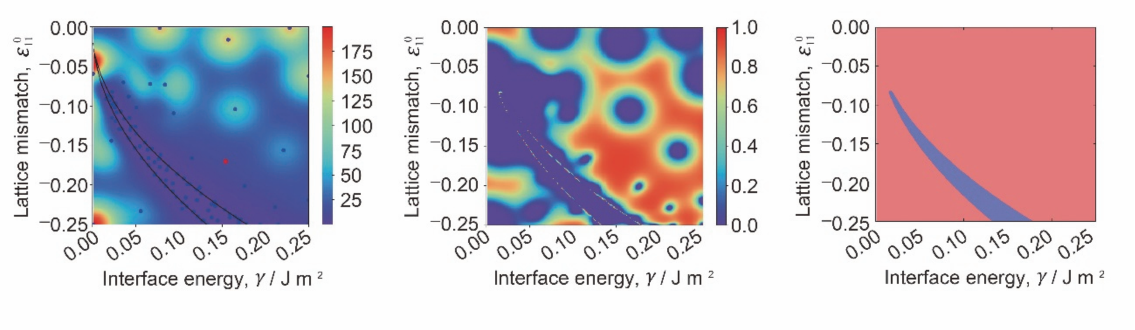

Supplementary Figure 1 Estimated LER with the proposed sampling strategy after 100 iterations. The threshold h is set to 1. The image on the left is the heatmap of $\mu(\boldsymbol{x}_i)$. Blue points represents the calculated points, and the red point is the one to be calculated in the subsequent iteration. Black lines are the boundary of the LER for $h = 1$. The image at the middle is the heatmap of IG. The image on the right is the predicted LER. Blue regions are $p(z_i = 1) \geq 0.5$, and red regions are $p(z_i = 1) < 0.5$.

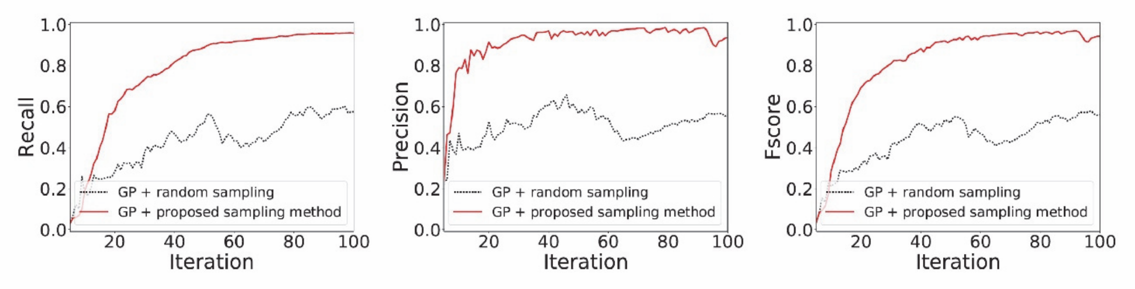

Supplementary Figure 2 Performance evaluation with $h = 1$. The image on the left is the ratio of the number of LER points that GP correctly identifies over the number of points in true LER (recall). The image at the middle is ratio of the number of LER points that GP correctly identifies over the number of points predicted as LER by GP (precision). The image on the right is the harmonic mean of recall and precision (F-score).
